# Diagnosis and mortality of emergency department patients in the North Denmark region

**DOI:** 10.1186/s12913-018-3361-x

**Published:** 2018-07-13

**Authors:** Morten Breinholt Søvsø, Sabina Bay Hermansen, Emil Færk, Tim Alex Lindskou, Marc Ludwig, Jørn Munkhof Møller, Jelena Jonciauskiene, Erika Frischknecht Christensen

**Affiliations:** 1 Centre for Prehospital and Emergency Research, Department of Clinical Medicine, Aalborg University, Søndre Skovvej 15, 9000 Aalborg, Denmark; 2 Emergency Department Hjørring, North Denmark Regional Hospital, Hjørring, Denmark; 3 Emergency Department & Trauma Centre, Aalborg University Hospital, Aalborg, Denmark; 4 Emergency Department Thy, North Denmark Regional Hospital, Thisted, Denmark; 5 Department of Anaesthesiology and Intensive Care, Aalborg University Hospital, Aalborg, Denmark

**Keywords:** Emergency department, Reorganization of healthcare, Delivery of healthcare, Non-specific diagnoses, Denmark, Mortality

## Abstract

**Background:**

Emergency departments handle a large proportion of acute patients. In 2007, it was recommended centralizing the Danish healthcare system and establishing emergency departments as the main common entrance for emergency patients. Since this reorganization, few studies describing the emergency patient population in this new setting have been carried out and none describing diagnoses and mortality. Hence, we aimed to investigate diagnoses and 1- and 30-day mortality of patients in the emergency departments in the North Denmark Region during 2014–2016.

**Methods:**

Population-based historic cohort study in the North Denmark Region (580,000 inhabitants) of patients with contact to emergency departments during 2014–2016. The study included patients who were referred by general practitioners (daytime and out-of-hours), by emergency medical services or who were self-referred.

Primary diagnoses (ICD-10) were retrieved from the regional Patient Administrative System. For non-specific diagnoses (ICD-10 chapter ‘Symptoms and signs’ and ‘Other factors’), we searched the same hospital stay for a specific diagnosis and used this, if one was given. We performed descriptive analysis reporting distribution and frequency of diagnoses. Moreover, 1- and 30-day mortality rate estimates were performed using the Kaplan-Meier estimator.

**Results:**

We included 290,590 patient contacts corresponding to 166 ED visits per 1000 inhabitants per year. The three most frequent ICD-10 chapters used were ‘Injuries and poisoning’ (38.3% *n* = 111,274), ‘Symptoms and signs’ (16.1% *n* = 46,852) and ‘Other factors’ (14.52% *n* = 42,195). Mortality at day 30 (95% confidence intervals) for these chapters were 0.86% (0.81–0.92), 3.95% (3.78–4.13) and 2.84% (2.69–3.00), respectively.

The highest 30-day mortality were within chapters ‘Neoplasms’ (14.22% (12.07–16.72)), ‘Endocrine diseases’ (8.95% (8.21–9.75)) and ‘Respiratory diseases’ (8.44% (8.02–8.88)).

**Conclusions:**

Patients in contact with the emergency department receive a wide range of diagnoses within all chapters of ICD-10, and one third of the diagnoses given are non-specific. Within the non-specific chapters, we found a 30-day mortality, surpassing several of the more organ specific ICD-10 chapters.

**Trial registration:**

Observational study - no trial registration was performed.

**Electronic supplementary material:**

The online version of this article (10.1186/s12913-018-3361-x) contains supplementary material, which is available to authorized users.

## Background

Emergency departments (ED) play a key role in the Danish healthcare system, handling a large proportion of all acute patients [1] – including smaller injuries, trauma and acute medical conditions.

In contrast to the ED setup in countries like the US [2], accessing the Danish EDs requires calling one of the acute healthcare services [3]. These healthcare services perform assessments of the health problem presented and based on this information determine which response is needed – including ED contact if necessary.

In 2007, the Danish Health Authority recommended centralizing the healthcare system in Denmark and establishing new and large EDs as a common entrance to the hospitals throughout the five Danish healthcare regions. Until then, emergency patients were admitted directly to a specific medical or surgical ward based on their presenting symptoms. The aim of these recommendations was to create greater opportunities for cooperation across medical specialties, contributing to faster treatment and avoiding unnecessary hospitalization [4].

The overall plan was that the approximately 40 hospitals with acute intake of patients in 2007 should reorganize into 21 larger EDs. By June 2016, 17 of these were up and running [1]. In line with the recommendations made, two EDs with a common intake of emergency patients were established in the North Denmark Region by 2013.

The introduction of EDs in Denmark was a major change in emergency care and organisation. Despite this, there is very limited research on the Danish ED patient population [5] and no studies investigating patient diagnoses. From prehospital studies, we know the pattern of diagnoses in the patients brought to hospital by emergency medical services (EMS) [6, 7], but the entire group of ED patients, including those referred from general practitioners (GPs) and self-referred patients are not well described regarding diagnoses and mortality.

Thus, the objective of this study was to investigate diagnoses and 1- and 30-day mortality of patients in the emergency departments in the North Denmark Region in 2014–2016.

## Methods

### Study design

We performed a population-based historic cohort study in the North Denmark Region of patient contacts to EDs in the region.

### Setting

The region has approximately 580,000 inhabitants and is primarily rural, with some larger urban areas. There are two larger hospitals in the region, the North Denmark Regional Hospital and Aalborg University Hospital, both have ED facilities and receive the majority of patients in the region. Accessing the EDs in the region requires calling either EMS through the national emergency number 1-1-2 or calling a GP (both daytime and out-of-hours). Time critical conditions requiring highly specialized interventions, such as ST-elevation myocardial infarction (STEMI) or stroke, are sent directly to treatment in specialized departments bypassing the ED. Psychiatric patients are received at a separate psychiatric emergency department.

The EMS use a criteria-based dispatch system to guide the call-handlers in the level of urgency and which response to send [8]. The call-handlers can also end the call by giving advice or referring the patient to a GP. Patients receiving an ambulance are usually brought to the ED, but if an EMS physician is involved, they can be treated and released on scene [9].

GPs can refer patients to the ED based on a consultation, home visit or just a telephone call - both during daytime and after hours [10]. They can contact the EMS if the patient needs an ambulance or non-emergency transportation (which is also part of the EMS). In certain situations, where transportation is not needed, patients arrive at the ED by their own means after consulting a GP.

A small proportion of patients do not contact this setup by phone, but simply show up at the ED.

### Study population and outcome

We defined the study population as patient contacts to the ED in a three-year period from the 1st of January 2014 to the 31st of December 2016 in the North Denmark Region. This included patients referred by a GP (daytime and out-of-hours), referred by EMS or self-referred. We only included patient contacts to the two larger hospitals in the region with EDs (North Denmark Regional Hospital and Aalborg University Hospital). Acute contacts to the smaller hospitals and ambulatory care functions in the region, which handle minor illness and injuries, but with limited medical specialties available, were not included. Patients admitted directly to a department (e.g. STEMI or stroke) were not included.

Patient contacts were included regardless whether they resulted in a hospital admission or not. Any patient contact in the North Denmark Region is registered in the Patient Administrative System (PAS), a regional patient registry providing data to the National Patient Registry [11]. We used PAS to obtain the patients’ unique civil registration number [12] and diagnoses for the contact as stated in the International Statistical Classification of Diseases, 10th Edition (ICD-10) [13]. We used the primary diagnosis the patient received. If this was a non-specific diagnosis (ICD-10 chapters ‘Symptoms and signs’ (‘Symptoms, signs and abnormal laboratory findings’) and ‘Other factors’ (‘Factors influencing health status and contact with health services’)), we used the first specific diagnosis given in relation to the hospital stay. If none was given, we used the non-specific primary diagnosis. All patient contacts during the study period were included, i.e., the same unique patient could have several ED contacts included.

Patient follow-up consisted of collecting vital status as to determine 1- and 30-day mortality. Mortality day 1 was defined as death on the same day or the day after the ED contact, as death registration is available only by date and not time of day. We chose this approach in order not to underestimate short-time mortality. We obtained information on vital status through the Danish Civil Registration System [12]. Vital status was not available if the patient was a tourist or had moved out of the region.

### Statistical analysis

Data were anonymized for statistical analysis. We used each patient contact to the ED as a unit in our analysis of data. If a patient was admitted or had a new contact within 2 hours of the prior, we considered this as one contact. Contacts or admissions at a later time were included as separate contacts. We performed descriptive analysis reporting the distribution of diagnoses according to the chapters of ICD-10 as frequencies and numbers. Stratified analyses were performed for sex and the age groups 0–10, 11–30, 31–60 and 61 years and older. Moreover, at individual diagnosis level the ten most frequently used diagnoses were reported as absolute numbers. We sorted the patient contacts into groups of 1, 2–5, 6–10 and 10 or more contacts and displayed the proportion of the total number of contacts shown in percent. We used the Kaplan-Meier estimator to report mortality rate estimates as percentages for day 1 and 30 with 95% confidence intervals (CI) and the cumulative number of deaths reported alongside. We performed additional sensitivity analysis including only the first patient contact to estimate mortality.

For the mortality estimates, 122 patient contacts were not included in the mortality analysis as they had diagnoses within the non-specific chapters e.g. ‘Other ill-defined and unspecified causes of mortality’ i.e. the patients were not alive when they received these diagnoses (see Additional file 1: Table S1 for detailed list of diagnoses). The contacts were included in all other analyses.

Statistical analyses were performed with Stata V.15.0/MP (Stata Corporation, College Station, Texas, USA).

## Results

**Fig. 1:**
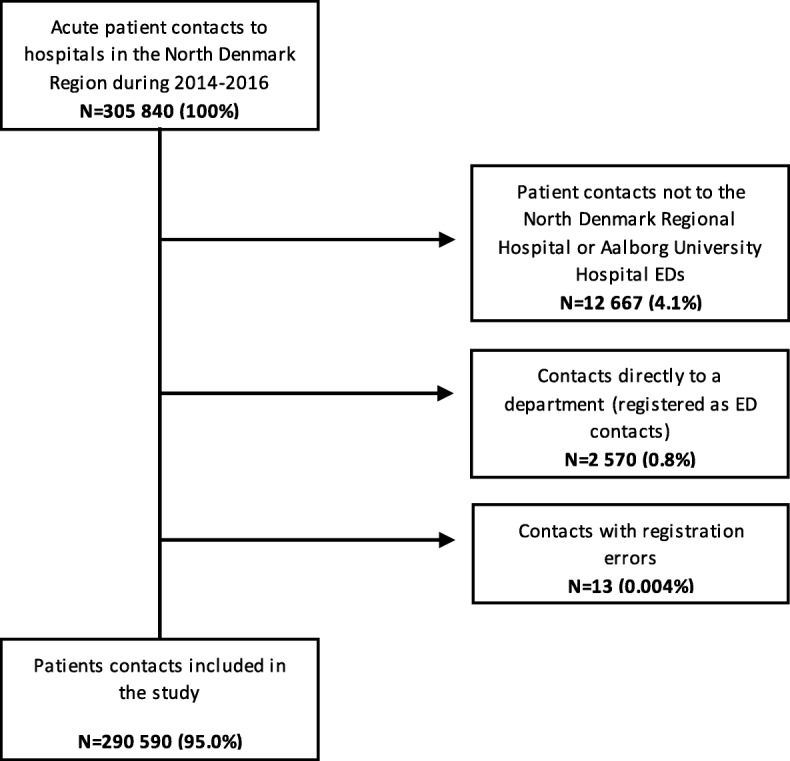
Flow chart for patient contacts included in the study

During the study period, there were 305,840 acute patient contacts to the hospitals in the North Denmark Region (Fig. 1). In 12,667 cases, patient contacts were not to the two large hospitals with EDs and therefore excluded. Moreover, 2570 patient contacts were registered as ED contacts, but were directly to a department and therefore excluded (674 patient contacts regarding pregnant women or women in labour and 1331 contacts regarding infants admitted to the neonatal department). Furthermore, 13 patient contacts were excluded due to registration errors (time of death registered before ED contact *n* = 7, diagnosis error *n* = 4 (perinatal diagnosis given at much older age) and missing diagnosis *n* = 2). Consequently, 290,590 patient contacts (166 contacts per 1000 inhabitants per year) were included in the study corresponding to 173,324 unique patients. Information on vital status was missing in 3.4% (10059) of all contacts, because the patient was a tourist or had moved out of the region.

The frequency of contacts for the 173,324 patients is displayed in Table 1. We found that one third of the patients had two or more contacts during the 3 years. A few extreme cases were found (seven patients with more than 50 contacts), but the majority of patients had one contact. For the entire study period, we found an admission rate of 88 admissions per 1000 inhabitants per year (admission defined as a hospital stay ≥24 h).

**Table 1:** Frequency of repeated contacts in the study period

Contact frequency	Number of patients	%
1	115,801	66.81
2–5	53,233	30.71
6–10	3555	2.05
> 10	735	0.42
Total	**173,324**	**100**

**Fig. 2:**
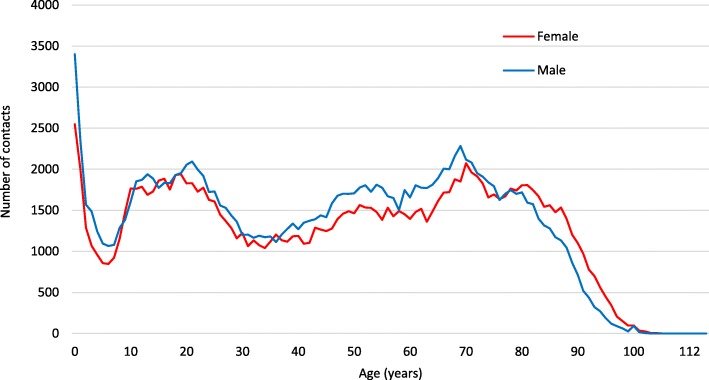
Patient age and sex distribution at time of ED contact

At time of ED contact, we found the age and sex distribution shown in Fig. 2. Mean age was 46.2 years (SD 27.1) with age peaks at 0 (< 1), 21 and 70 years with 0 (< 1) as the single most frequent age in the entire population. 48.5% of the population were female.

**Fig. 3:**
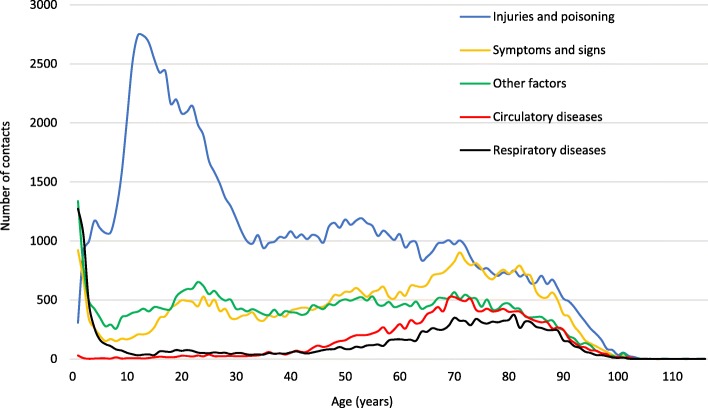
Top five most frequent ICD-10 chapters and patient age distribution at time of ED contact

Figure 3 shows the five most frequent ICD-10 chapters and corresponding distribution of patient age at time of ED contact.

‘Injuries and poisoning’ (‘Injury, poisoning and certain other consequences of external causes’) was the most common chapter used with 64 contacts per 1000 inhabitants per year. The chapter contained a large group of younger patients and we found that with increasing age, fewer patients received diagnoses within the chapter.

With 27 contacts per 1000 inhabitants per year ‘Symptoms and signs‘, was the second most frequent chapter. Within this chapter, there was a spike for small children and then again a gradual increase in the older ages.

‘Other factors’ was the third most frequent chapter (24 contacts per 1000 inhabitants per year) and evenly distributed across ages, although in the chapter, there was also a spike for the small children.

‘Circulatory diseases’ (‘Diseases of the circulatory system’) and ‘Respiratory diseases (‘Diseases of the respiratory system’) were the fourth and fifth most frequent chapters with almost the same proportion of the entire population (both corresponding to 9 contacts per 1 000 inhabitants per year). ‘Circulatory diseases’ were distributed primarily among the older part of the population. In contrast, we find a large spike in frequency for the very young children within ‘Respiratory diseases’ and a notable increase as age increases.

The full distribution of ICD-10 chapters sorted by age groups (0–10, 11–30, 31–60 and 61+) and sex is displayed in Additional file 2: Table S2 and Additional file 3: Table S3.

Overall, more than two thirds of all patient contacts resulted in diagnoses within the three ICD-10 chapters ‘Injuries and poisoning’, ‘Symptoms and signs’ and ‘Other factors’. Moreover, almost one third of the contacts received a diagnosis within the latter two non-specific chapters.

Table 2 shows the 1- and 30-day mortality estimates, sorted by ICD-10 chapters (see Additional file 4: Table S4 for full table).

**Table 2:** Hospital diagnoses (ICD-10 chapters) sorted by cumulative mortality of 290,468 patient contacts to the EDs of the North Denmark Regional Hospital and Aalborg University Hospital during 2014–2016

	Cumulative number of deaths day 1	1-day mortality percent (95% CI)	Cumulative number of deaths day 30	30-day mortality Percent (95% CI)
ICD-10 Chapter	**N**	**%**	**N**	**%**
Symptoms and signs	174	0.37(0.32–0.43)	1845	3.95(3.78–4.13)
Respiratory diseases	261	1.65(1.46–1.86)	1335	8.44(8.02–8.88)
Circulatory diseases	463	2.82(2.58–3.08)	1234	7.51(7.12–7.92)
Other factors	235	0.56(0.49–0.63)	1198	2.84(2.69–3.00)
Injuries and poisoning	68	0.06(0.05–0.08)	958	0.86(0.81–0.92)
Digestive diseases	78	0.51(0.41–0.64)	524	3.44(3.16–3.74)
Endocrine diseases	31	0.59(0.41–0.83)	472	8.95(8.21–9.75)
Infections	110	1.55(1.29–1.87)	468	6.61(6.06–7.22)
Other chapters	19	0.09(0.06–0.14)	261	1.20(1.06–1.35)
Genitourinary diseases	14	0.25(0.15–0.41)	211	3.69(3.24–4.22)
Blood diseases	14	0.69(0.41–1.15)	142	6.95(5.93–8.14)
Neoplasms	14	1.61(0.95–2.70)	124	14.22(12.07–16.72)
Total	**1481**	**0.51(0.48–0.54)**	**8772**	**3.02(2.96–3.08)**

Highest 1-day mortality in percentage was found among contacts receiving diagnoses within the chapters (in descending order) ‘Circulatory diseases’ (2.82%), ‘Respiratory diseases’ (1.65%), ‘Neoplasms’ (‘Neoplasms’) (1.61%) and ‘Infections’ (‘Certain infectious and parasitic diseases’) (1.55%). The remaining chapters each had 1-day mortality below 1%.

At 30 days, the highest mortality in percentage was within the chapters ‘Neoplasms’ (14.22%), ‘Endocrine diseases‘ (‘Endocrine, nutritional and metabolic diseases’) (8.95%), ‘Respiratory diseases’ (8.44%), ‘Circulatory diseases’ (7.51%), ‘Blood diseases‘ (‘Diseases of the blood and blood-forming organs and certain disorders involving the immune mechanism’) (6.95%), ‘Infections’ (6.61%) and ‘Symptoms and signs’ (3.95%). The remaining chapters each had a 30-day mortality below 4%. The tables are in order of cumulative deaths on day 30.

Table 3 shows the most frequently used individual diagnoses in the entire patient population. Looking at the ten most frequent individual diagnoses, we find that the majority are from the two non-specific chapters ‘Symptoms and signs’ or ‘Other factors’. The single most frequent diagnosis in the ED was ‘Observation for suspected disease or condition, unspecified’ (DZ039) used more than every 20th contact.

**Table 3:** The ten most frequently used ICD-10 diagnosis for patient ED contacts during 2014–2016 and corresponding cumulative deaths and mortality estimates sorted by frequency

ICD-10 diagnosis	N	%	Cumulative number of deaths day 1	1-day mortality percent (95% CI)	Cumulative number of deaths day 30	30-day mortality percent (95% CI)
DZ039 Observation for suspected disease or condition, unspecified	16,583	5.71	171	1.03(0.89–1.20)	736	4.44(4.14–4.76)
DR100 Acute abdomen	7293	2.51	17	0.23(0.14–0.37)	175	2.40(2.07–2.78)
DS934 Sprain and strain of ankle	7107	2.45	0	–	9	0.24(0.13–0.47)
DZ768 Persons encountering health services in other specified circumstances	6533	2.25	17	0.26(0.16–0.42)	73	1.12(0.89–1.40)
DZ038 Observation for other suspected diseases and conditions	5293	1.82	10	0.19(0.10–0.35)	114	2.15(1.80–2.58)
DR074 Chest pain, unspecified	4899	1.69	13	0.27(0.15–0.46)	57	1.16(0.90–1.51)
DJ189 Pneumonia, unspecified	4581	1.58	64	1.40(1.10–1.78)	525	11.46(10.57–12.42)
DR060 Dyspnoea	3871	1.33	46	1.19(0.89–1.58)	495	12.79(11.77–13.88)
DS525 Fracture of lower end of radius	3697	1.27	0	–	9	0.24(0.13–0.47)
DS060 Concussion	3393	1.16	3	0.09(0.03–0.27)	26	0.77(0.52–1.12)

## Discussion

### Key results

This population-based historic cohort study showed that patients with contact to two Danish emergency departments received a large variety of diagnoses within all ICD-10 chapters. As the EDs handle injuries as cuts, fractures and trauma, not surprisingly the most frequent chapter used was ‘Injuries and poisoning’. This accounted for more than one-third of all the contacts.

More interestingly, we found that almost one third of the contacts received a diagnosis within the two non-specific chapters ‘Symptoms and signs’ or ‘Other factors’. Although we looked for specific diagnoses in relation to all contacts, there was a large proportion of non-specific diagnoses. This is underlined by the single most used diagnosis being ‘Observation for suspected disease or condition, unspecified (DZ039)’.

Moreover, we found that 30-day mortality for the non-specific chapters surpassed several of the more organ-specific chapters. At day 1, the highest mortality was for ‘Circulatory diseases’ whereas the remaining chapters had a 1-day mortality below 2%.

The highest 30-day mortality was reported for ‘Neoplasms’. Chapters ‘Endocrine diseases’, ‘Respiratory diseases’ and ‘Circulatory diseases’ also had relatively high 30-day mortality.

We performed additional sensitivity analysis including only the first patient contact to estimate mortality (see Additional file 5: Table S5), which only lead to a slight change of order within the chapters with the highest mortality.

An interesting group of patients in the study were children < 1 years. This was the single most frequent age group in contact with the ED. Consequently, it would be interesting to investigate this group more thoroughly regarding diagnoses, mode of referral and parents’ perspective.

### Strengths and weaknesses of the study

One major strength of the study is its population-based design. Thus, it includes all patient contacts to the EDs in this region, which minimizes selection bias and allows a strong follow-up.

Another major strength is the linkage of patient contacts to data registries through the unique civil registration number of each patient.

In the present study, we performed our analysis from the perspective of the ED, i.e. the main unit in our data was an ED contact. This meant the same patient could be included several times and the mortality estimates are therefore based on each patient contact. We decided on this setup, as this is the reality in an ED: every time the patient has a new contact, a new assessment of the patient is performed. We chose to complement this analysis with mortality estimates based only on the first contact the patient had to the ED, hence looking at it from the patient’s perspective. We considered it a strength, that we performed both analyses.

In the present study setup, it is not possible to elaborate on the reasons behind the non-specific diagnosis within the ED population. We do not know the extent of self-referred patients or the mode of referral in general. Similarly, information on GP use (daytime and out-of-hours) before and after ED contact and more extensive history of admissions and readmissions would contribute to understanding this patient group and the ED population in general.

The mortality within the non-specific patient group in the present study could be explained by comorbidity, which we did not investigate. With this, we would have been able to describe any differences in comorbidity between relevant patient groups e.g. high vs. low mortality or non-specific vs. specific diagnoses. Including this would strengthen future studies.

The mortality could also be explained by a number of life-threatening diagnoses within the two non-specific chapters – such as ‘respiratory arrest (DR092)’ and ‘cardiorespiratory failure (DR092A)’ albeit these represent very few cases (total *n* = 21). In the mortality rate estimation of the non-specific chapters, we excluded patient contacts with diagnoses concerning unspecified causes of mortality i.e. the patients are not alive at time of diagnosis (*n* = 122), as these cases would otherwise lead to an overestimation of mortality within the chapters.

Another minor weakness related to the mortality data could occur if a patient moved out of the region during the study. Data on vital status were limited to patients in the North and Central Denmark Region, which meant patients moving elsewhere and tourists, will have incomplete mortality reporting.

### Other studies

Most international studies concerning patients in the ED revolve around patients with many contacts to the EDs and their characteristics [14, 15], whereas fewer studies have described the entire ED population. No study has described the emergency patient population regarding diagnosis and mortality in the new Danish ED setting.

A large part of ED studies originate from the US where the gatekeeper function of GPs is absent. This function aims to ensure that patients in need of more specialized care can access secondary healthcare facilities, but also that patients are guided to primary care, when this is sufficient. Some patients still by-pass this setup, a well-known issue in countries with similar healthcare setups [16, 17]. Considering the differences in setup, we primarily compared our study to studies from countries with similar healthcare organization e.g. Norway and Iceland.

Carter-Storch et al. published a Danish cross-sectional study based on data from 2010 [5] aiming to categorize complaints and symptoms of admitted ED patients into major groups. As a secondary result, they found the following distribution of patients: 49% medical, 31% surgical, 15% orthopaedic and 5% vascular surgical. This study included only admitted patients and did not use discharge diagnoses, but collected data regarding complaints and symptoms from the referring doctor or the patients, making it difficult to compare with the present study.

A Danish-American study by Dalgaard et al. [18] investigated and described the population in an ED in Boston, USA, and concluded that patients received non-specific diagnoses in 26.5% of the cases. The distribution of the remaining chapters were also in good agreement with our findings (albeit the classification used was ICD-9), which is interesting as the gatekeeper function of the GP was absent. Around two thirds of the patients presenting to the ED were discharged without admission - one could hypothesize that a part of these patients could have been seen by a GP instead.

In a Norwegian prospective study from 2014 [19], Langlo et al. assigned International Classification of Primary Care (ICPC-2) codes for presenting complaints and symptoms of 3163 patients in an ED during a 2 month period. They found the most frequent ICPC-2 classifications chapters to be general and unspecified 37%, digestive 19%, respiratory 12%, neurological 12% and musculoskeletal 6%. Although, the ICPC-2 and ICD-10 classifications differ, there are some similarities. Non-specific chapters also account for almost one third in our study and diagnoses of digestive and respiratory diseases are likewise quite frequently given. During daytime, the Norwegian ED has a separate clinic handling injuries and minor traumas, which explains the dissimilarity in the findings.

Vest-Hansen et al. investigated 264,265 acute hospital admissions to medical wards (not EDs) during 2010 in a large Danish population-based observational study [20] and described the pattern of diagnosis for this group. The most frequent ICD-10 chapters were non-specific (28.7%), circulatory diseases (19.3%), infections (15.5%) and injuries and poisoning (6.3%) (adverse effects, intracranial injury and poisoning by psychotropic drugs were the most frequent diagnoses in this chapter)*.* The study included only patients admitted to medical wards, which could explain the higher proportion of circulatory diseases and infections, whereas orthopaedic patients and other surgical patients were not included.

Perhaps the non-specific patient group needs further attention. This is somewhat underlined by a Danish study where Hansen et al. [21] followed 409 patients with initial non-specific diagnoses (DZ03) in an acute medical admission unit. In the patient group discharged with non-specific diagnoses, 28% were readmitted within 30 days of discharge and 76% of these received a more specific diagnosis.

An interesting American study from 2014 by Raven et al. [22] investigated and found only very limited correspondence between discharge diagnoses from the ED compared to the patients’ presenting complaints in the ED. However, this study used ICD-9, which is less updated and contains much fewer diagnoses than ICD-10 [23], which could have meant that the diagnoses used were less specific. In addition, the settings for these studies were quite different. The Danish setting had a gatekeeper function in the form of a GP (both during work hours and after hours). Thus, many of the ED patients have been assessed by a GP before their ED contact, which could have led to a better correspondence between complaints and discharge diagnoses.

In an Icelandic descriptive study from 2006 by Gunnarsdottir et al. [24] investigated patients discharged from the ED without admission. Patients within the psychiatric, paediatric, gynaecology and obstetrics fields were not included. The study included the years 1995–2001. In the last year included, they too found the non-specific ICD-10 chapters to account for 29.4% of the contacts followed by circulatory diseases (16.5%) and diseases of the genitourinary tract (10.8%).

One of the key result of the present study is the mortality within the non-specific chapters. In a recent study of 148,757 patients from the same Danish region, Christensen et al. [6] found that patients receiving non-specific diagnoses after being brought to hospital by ambulance had a 30-day mortality of 4.3%. This correlates well with our findings as did the distribution of the most frequent ICD-10 diagnosis chapters (injury and poisoning 30.0%, symptoms and abnormal findings, not elsewhere classified 17.5% and factors influencing health status and contact with health services 14.1%).

### Interpretation

This study shows that ED patients receive a large variety of diagnoses across all ICD-10 chapters, but the majority of patient contacts leads to a diagnosis within the ‘injuries and poisoning’ or non-specific ICD-10 diagnosis chapters. Similar large proportions of non-specific diagnoses have been found in various other studies from comparable healthcare settings.

Several reasons could be at play as to why we find this large group of non-specific diagnoses. On one hand, we see an increasing overload of the EDs, less available beds for admissions and consequential demand for handling more patients in the ED, which could lead to more use of non-specific diagnoses. On the other hand, a large proportion of the patient population is characterized by chronic disease and multi-morbidity leading to complex symptomatology that does not fit well in the confinement of the diagnoses of the ICD-10.

Only very few studies describing the ED populations exist and the results of this first description of a Danish ED population’s diagnosis and mortality could interest public healthcare planners and policy experts, when making administrative decisions.

Moreover, the Danish EDs have changed in recent years and this study elucidates some of the implications following the reorganization i.e. changes in the patient population.

Taken into consideration that a Danish emergency medicine medical specialty has emerged perhaps the education planners may find the study relevant.

## Additional files

Additional file 1: **Table S1.** List of ICD-10 diagnoses not included in mortality estimates (DOCX 12 kb)

Additional file 2: **Table S2.** Age-separated distribution of hospital diagnoses (ICD-10 chapters) for 290,590 patient contacts at the EDs at the North Denmark Regional Hospital and Aalborg University Hospital during 2014–2016. (DOCX 17 kb)

Additional file 3: **Table S3.** Sex-separated distribution of hospital diagnoses (IC-10 chapters) for 290,590 patient contacts at the EDs at the North Denmark Regional Hospital and Aalborg University Hospital during 2014–2016. (DOCX 15 kb)

Additional file 4: **Table S4.** Hospital diagnoses (ICD-10 chapters) sorted by cumulative mortality of 290,468 patient contacts at the EDs of the North Denmark Regional Hospital and Aalborg University Hospital during 2014–2016. (DOCX 15 kb)

Additional file 5: **Table S5.** Hospital diagnoses (ICD-10 chapters) sorted by cumulative number of deaths when using first contact of 290,468 patient contacts at the EDs of the North Denmark Region during 2014–2016. (DOCX 15 kb)

## Abbreviations

ED: Emergency departments
EMS: Emergency medical services
GP: General practitioner
ICD-10: International Statistical Classification of Diseases, 10th Edition
ICPC-2: International Classification of Primary Care, 2nd edition
PAS: Patient Administrative System
STEMI: ST-Elevation Myocardial Infarction
