# Supplementary material for: Diagnosis and mortality of emergency department patients in the North Denmark region
Source: BMC Health Serv Res. 2018 Jul 13;18:548. doi: 10.1186/s12913-018-3361-x (PMC6044093; doi:10.1186/s12913-018-3361-x)
Supplement: Supplementary file 1 — Table S1. List of ICD-10 diagnoses not included in mortality estimates (DOCX 12 kb) [file 12913_2018_3361_MOESM1_ESM.docx]

**Supplementary Table 1**

| DR99 Other ill-defined and unspecified causes of mortality |
| --- |
| DR99 Special circumstances regarding death |
| DR991 Brain death according to the Danish Health act § 176 |
| DR992 Cardiac death according to the Danish Health act § 176 |

**List of ICD-10 diagnoses not included in mortality estimates**
