# Supplementary material for: Diagnosis and mortality of emergency department patients in the North Denmark region
Source: BMC Health Serv Res. 2018 Jul 13;18:548. doi: 10.1186/s12913-018-3361-x (PMC6044093; doi:10.1186/s12913-018-3361-x)
Supplement: Supplementary file 2 — Table S2. Age-separated distribution of hospital diagnoses (ICD-10 chapters) for 290,590 patient contacts at the EDs at the North Denmark Regional Hospital and Aalborg University Hospital during 2014–2016. (DOCX 17 kb) [file 12913_2018_3361_MOESM2_ESM.docx]

**Supplementary Table 2**

| ICD-10 chapter, separated by age groups (years) | 0-10 | | 11-30 | | 31-60 | | 61+ | | Total | |
| --- | --- | --- | --- | --- | --- | --- | --- | --- | --- | --- |
|  | **N** | **%** | **N** | **%** | **N** | **%** | **N** | **%** | **N** | **%** |
| Injuries and poisoning | 14 013 | 43.19 | 39 802 | 57.96 | 31 782 | 37.97 | 25 677 | 24.27 | 111 274 | 38.29 |
| Symptoms and signs | 3 393 | 10.46 | 7 808 | 11.37 | 14 199 | 16.96 | 21 452 | 20.28 | 46 852 | 16.12 |
| Other factors | 5 326 | 16.42 | 9 998 | 14.56 | 13 260 | 15.84 | 13 611 | 12.87 | 42 195 | 14.52 |
| Circulatory diseases | 82 | 0.25 | 401 | 0.58 | 3 936 | 4.70 | 12 010 | 11.35 | 16 429 | 5.65 |
| Respiratory diseases | 3 697 | 11.40 | 1 055 | 1.54 | 2 482 | 2.97 | 8 584 | 8.12 | 15 818 | 5.44 |
| Digestive diseases | 841 | 2.59 | 2 310 | 3.36 | 5 349 | 6.39 | 6 731 | 6.36 | 15 231 | 5.24 |
| Musculoskeletal diseases | 476 | 1.47 | 1 091 | 1.59 | 2 669 | 3.19 | 2 995 | 2.83 | 7 231 | 2.49 |
| Infections | 1 646 | 5.07 | 841 | 1.22 | 1 364 | 1.63 | 3 224 | 3.05 | 7 075 | 2.43 |
| Genitourinary diseases | 535 | 1.65 | 1 007 | 1.47 | 1 506 | 1.80 | 2 664 | 2.52 | 5 712 | 1.97 |
| Endocrine diseases | 549 | 1.69 | 529 | 0.77 | 1 033 | 1.23 | 3 162 | 2.99 | 5 273 | 1.81 |
| Mental disorders | 26 | 0.08 | 1 642 | 2.39 | 2 448 | 2.92 | 955 | 0.90 | 5 071 | 1.75 |
| Neurological diseases | 326 | 1.00 | 757 | 1.10 | 1 444 | 1.73 | 1 733 | 1.64 | 4 260 | 1.47 |
| Skin diseases | 356 | 1.10 | 1 071 | 1.56 | 1 416 | 1.69 | 842 | 0.80 | 3 685 | 1.27 |
| Blood diseases | 190 | 0.59 | 132 | 0.19 | 330 | 0.39 | 1 391 | 1.32 | 2 043 | 0.70 |
| Neoplasms | 231 | 0.71 | 87 | 0.13 | 143 | 0.17 | 411 | 0.39 | 872 | 0.30 |
| Ear diseases | 190 | 0.59 | 41 | 0.06 | 175 | 0.21 | 212 | 0.20 | 618 | 0.21 |
| Perinatal diseases | 433 | 1.33 | 0 | 0 | 0 | 0 | 0 | 0 | 433 | 0.15 |
| Eye diseases | 46 | 0.14 | 69 | 0.10 | 141 | 0.17 | 107 | 0.10 | 363 | 0.12 |
| Congenital diseases | 88 | 0.27 | 28 | 0.04 | 23 | 0.03 | 16 | 0.02 | 155 | 0.05 |
| Total | **32 444** | **100** | **68 669** | **100** | **83 700** | **100** | **105 777** | **100** | **290 590** | **100** |

**Age-separated distribution of hospital diagnoses (ICD-10 chapters) for 290 590 patient contacts at the EDs at the North Denmark Regional Hospital and Aalborg University Hospital during 2014-2016.**
