# Supplementary material for: Diagnosis and mortality of emergency department patients in the North Denmark region
Source: BMC Health Serv Res. 2018 Jul 13;18:548. doi: 10.1186/s12913-018-3361-x (PMC6044093; doi:10.1186/s12913-018-3361-x)
Supplement: Supplementary file 3 — Table S3. Sex-separated distribution of hospital diagnoses (IC-10 chapters) for 290,590 patient contacts at the EDs at the North Denmark Regional Hospital and Aalborg University Hospital during 2014–2016. (DOCX 15 kb) [file 12913_2018_3361_MOESM3_ESM.docx]

**Supplementary Table 3**

|  | Female | | Male | | Total |
| --- | --- | --- | --- | --- | --- |
| ICD-10 chapter, separated by sex | **N** | **%** | **N** | **%** | **N** |
| Injuries and poisoning | 53 659 | 48.22 | 57 615 | 51.78 | 111 274 |
| Symptoms and signs | 24 157 | 51.56 | 22 695 | 48.44 | 46 852 |
| Other factors | 20 747 | 49.17 | 21 448 | 50.83 | 42 195 |
| Circulatory diseases | 6 958 | 42.35 | 9 471 | 57.65 | 16 429 |
| Respiratory diseases | 7 459 | 47.16 | 8 359 | 52.84 | 15 818 |
| Digestive diseases | 7 422 | 48.73 | 7 809 | 51.27 | 15 231 |
| Musculoskeletal diseases | 3 575 | 49.44 | 3 656 | 50.56 | 7 231 |
| Infections | 3 249 | 45.92 | 3 826 | 54.08 | 7 075 |
| Genitourinary diseases | 3 242 | 56.76 | 2 470 | 43.24 | 5 712 |
| Endocrine diseases | 2 745 | 52.06 | 2 528 | 47.94 | 5 273 |
| Mental disorders | 2 023 | 39.89 | 3 048 | 60.11 | 5 071 |
| Neurological diseases | 2 117 | 49.69 | 2 143 | 50.31 | 4 260 |
| Skin diseases | 1 658 | 44.99 | 2 027 | 55.01 | 3 685 |
| Blood diseases | 958 | 46.89 | 1 085 | 53.11 | 2 043 |
| Neoplasms | 389 | 44.61 | 483 | 55.39 | 872 |
| Ear diseases | 313 | 50.65 | 305 | 49.35 | 618 |
| Perinatal diseases | 175 | 40.42 | 258 | 59.58 | 433 |
| Eye diseases | 153 | 42.15 | 210 | 57.85 | 363 |
| Congenital diseases | 57 | 36.77 | 98 | 63.23 | 155 |
| Total | **141 056** | **48.54** | **149 534** | **51.46** | **290 590** |

**Sex-separated distribution of hospital diagnoses (IC-10 chapters) for 290 590 patient**

**contacts at the EDs at the North Denmark Regional Hospital and Aalborg University**

**Hospital during 2014-2016.**
