# Supplementary material for: Diagnosis and mortality of emergency department patients in the North Denmark region
Source: BMC Health Serv Res. 2018 Jul 13;18:548. doi: 10.1186/s12913-018-3361-x (PMC6044093; doi:10.1186/s12913-018-3361-x)
Supplement: Supplementary file 4 — Table S4. Hospital diagnoses (ICD-10 chapters) sorted by cumulative mortality of 290,468 patient contacts at the EDs of the North Denmark Regional Hospital and Aalborg University Hospital during 2014–2016. (DOCX 15 kb) [file 12913_2018_3361_MOESM4_ESM.docx]

**Supplementary table 4**

|  | Cumulative number of deaths day 1 | 1-Day mortality percent (95% CI) | Cumulative number of deaths day 30 | 30-day mortality  Percent (95% CI) |
| --- | --- | --- | --- | --- |
| ICD-10 Chapter | **N** | **%** | **N** | **%** |
| Symptoms and signs | 174 | 0.37(0.32-0.43) | 1 845 | 3.95(3.78-4.13) |
| Respiratory diseases | 261 | 1.65(1.46-1.86) | 1 335 | 8.44(8.02-8.88) |
| Circulatory diseases | 463 | 2.82(2.58-3.08) | 1 234 | 7.51(7.12-7.92) |
| Other factors | 235 | 0.56(0.49-0.63) | 1 198 | 2.84(2.69-3.00) |
| Injuries and poisoning | 68 | 0.06(0.05-0.08) | 958 | 0.86(0.81-0.92) |
| Digestive diseases | 78 | 0.51(0.41-0.64) | 524 | 3.44(3.16-3.74) |
| Endocrine diseases | 31 | 0.59(0.41-0.83) | 472 | 8.95(8.21-9.75) |
| Infections | 110 | 1.55(1.29-1.87) | 468 | 6.61(6.06-7.22) |
| Genitourinary diseases | 14 | 0.25(0.15-0.41) | 211 | 3.69(3.24-4.22) |
| Blood diseases | 14 | 0.69(0.41-1.15) | 142 | 6.95(5.93-8.14) |
| Neoplasms | 14 | 1.61(0.95-2.70) | 124 | 14.22(12.07-16.72) |
| Musculoskeletal diseases | 7 | 0.10(0.05-0.20) | 101 | 1.40(1.15-1.69) |
| Mental disorders | 5 | 0.10(0.04-0.24) | 59 | 1.16(0.90-1.50) |
| Neurological diseases | 7 | 0.16(0.08-0.34) | 56 | 1.31(1.01-1.70) |
| Skin diseases | 0 | 0 | 41 | 1.11(0.82-1.51) |
| Ear diseases | 0 | 0 | 2 | 0.32(0.08-1.29) |
| Congenital diseases | 0 | 0 | 1 | 0.65(0.09-4.49) |
| Eye diseases | 0 | 0 | 1 | 0.28(0.04-1.94) |
| Perinatal diseases | 0 | 0 | 0 | 0 |
| Total | **1 481** | **0.51(0.48-0.54)** | **8 772** | **3.02(2.96-3.08)** |

**Hospital diagnoses (ICD-10 chapters) sorted by cumulative mortality of 290 468 patient contacts at the EDs of the North Denmark**

**Regional Hospital and Aalborg University Hospital during 2014-2016.**
