# Supplementary material for: Diagnosis and mortality of emergency department patients in the North Denmark region
Source: BMC Health Serv Res. 2018 Jul 13;18:548. doi: 10.1186/s12913-018-3361-x (PMC6044093; doi:10.1186/s12913-018-3361-x)
Supplement: Supplementary file 5 — Table S5. Hospital diagnoses (ICD-10 chapters) sorted by cumulative number of deaths when using first contact of 290,468 patient contacts at the EDs of the North Denmark Region during 2014–2016. (DOCX 15 kb) [file 12913_2018_3361_MOESM5_ESM.docx]

**Supplementary Table 5**

|  | Cumulative number of deaths day 1 | 1-Day mortality percent (95% CI) | Cumulative number of deaths day 30 | 30-day mortality  Percent (95% CI) |
| --- | --- | --- | --- | --- |
| ICD-10 Chapter | **N** | **%** | **N** | **%** |
| Circulatory diseases | 259 | 2.76(2.45-3.11) | 638 | 6.81(6.31-7.33) |
| Symptoms and signs | 54 | 0.22(0.17-0.28) | 556 | 2.23(2.06-2.42) |
| Other factors | 111 | 0.45(0.37-0.54) | 464 | 1.88(1.72-2.05) |
| Respiratory diseases | 86 | 1.13(0.92-1.39) | 436 | 5.73(5.23-6.27) |
| Injuries and poisoning | 37 | 0.05(0.04-0.07) | 416 | 0.56(0.51-0.62) |
| Digestive diseases | 29 | 0.32(0.22-0.46) | 198 | 2.20(1.91-2.52) |
| Infections | 44 | 1.12(0.84-1.50) | 176 | 4.49(3.88-5.18) |
| Endocrine diseases | 19 | 0.74(0.47-1.16) | 158 | 6.17(5.31-7.18) |
| Neoplasms | 9 | 2.13(1.12-4.06) | 66 | 15.64(12.50-19.47) |
| Genitourinary diseases | 3 | 0.09(0.03-0.28) | 66 | 1.98(1.56-2.51) |
| Musculoskeletal diseases | 1 | 0.02(0.00-0.16) | 38 | 0.88(0.64-1.20) |
| Blood diseases | 3 | 0.35(0.11-1.08) | 36 | 4.18(3.03-5.75) |
| Neurological disease | 2 | 0.08(0.02-0.34) | 23 | 0.98(0.65-1.47) |
| Mental disorders | 1 | 0.04(0.01-0.30) | 17 | 0.72(0.45-1.15) |
| Skin diseases | 0 | - | 14 | 0.64(0.38-1.08) |
| Eye diseases | 0 | - | 1 | 0.40(0.06-2.81) |
| Ear diseases | 0 | - | 0 | - |
| Perinatal diseases | 0 | - | 0 | - |
| Congenital diseases | 0 | - | 0 | - |
| Total | **658** | **0.38(0.35-0.41)** | **3 303** | **1.91(1.84-1.97)** |

**Hospital diagnoses (ICD-10 chapters) sorted by cumulative number of deaths when using first contact of 290 468**

**patient contacts at the EDs of the North Denmark Region during 2014-2016.**
